# Distinct Responses of Gut Microbiota to Jian-Pi-Yi-Shen Decoction Are Associated With Improved Clinical Outcomes in 5/6 Nephrectomized Rats

**DOI:** 10.3389/fphar.2020.00604

**Published:** 2020-05-06

**Authors:** Lin Zheng, Shuo Chen, Fochang Wang, Shiying Huang, Xinhui Liu, Xilan Yang, Haokui Zhou, Guo-Ping Zhao, Mingjing Luo, Shunmin Li, Jianping Chen

**Affiliations:** ^1^Shenzhen Key Laboratory of Hospital Chinese Medicine Preparation, Shenzhen Traditional Chinese Medicine Hospital, The Fourth Clinical Medical College of Guangzhou University of Chinese Medicine, Shenzhen, China; ^2^Shenzhen Institute of Synthetic Biology, Shenzhen Institutes of Advanced Technology, Chinese Academy of Sciences, Shenzhen, China; ^3^CAS Key Laboratory of Quantitative Engineering Biology, Shenzhen Institutes of Advanced Technology, Shenzhen, China; ^4^Department of Nephrology, Shenzhen Traditional Chinese Medicine Hospital, The Fourth Clinical Medical College of Guangzhou University of Chinese Medicine, Shenzhen, China

**Keywords:** gut microbiota, Jian-Pi-Yi-Shen decoction, piperazine ferulate, chronic kidney disease, 5/6 nephrectomized rats

## Abstract

Gut dysbiosis contributes to the development and progression of chronic kidney disease (CKD) and its complications. However, the effect of drugs on the gut microbiota of CKD patients and its influence on treatment outcomes remains to be explored. Here, we assessed whether the response of gut microbiota to the traditional Chinese medicine Jian-Pi-Yi-Shen (JPYS) decoction differed from that to piperazine ferulate (PF), a kidney-targeted drug, by 16S rDNA sequencing, and whether the difference could be linked with drug-specific clinical outcomes. We showed that both JPYS and PF improved renal function, but only JPYS was able to restore the blood reticulocyte counting and serum calcium level in CKD rats. We also found that weighted UniFrac beta-diversity of the gut microbiome of the JPYS treated rats was significantly different from that of PF. Microbiome markers of drug-specific response were identified and subjected to correlation network analysis, together with clinical parameters and KEGG pathways. Among the microbiome markers of CKD, *Corynebacterium* was found to form a network hub that was closely correlated with the JPYS responder *Enterococcus*, suggesting a potential indirect impact of JPYS on *Corynebacterium via* interspecies interactions. We also identified two network hubs of the PF responder *Blautia* and the JPYS-only marker *Coprococcus*, which were connected with many genera and clinical parameters. They might serve as keystone taxa driving the response of gut microbiota to the drugs and influence host outcomes. Moreover, the JPYS-only marker *Clostridium_XIVb* was found to be connected to many pathways that are associated with CKD progression and might account for the improved outcomes in the JPYS treated rats. At last, the identified keystone markers of drug response were validated by qPCR for their differential abundance between CKD and the two drugs. Taken together, our study revealed that the responses of gut microbiota to JPYS were distinct from that to PF, and pinpointed drug-specific keystone microbiome markers closely correlated to clinical parameters, which could serve as candidate microbiome targets for further studies on their roles in medicating the drug efficacy of TCM in CKD.

## Introduction

Chronic kidney disease (CKD) is characterized by a progressive loss of renal function and is associated with increased risk of multiple complications, including anemia, mineral bone diseases and cardiovascular diseases. Recent studies have revealed the critical roles played by gut microbiota in the development and progression of CKD (Jiang et al., 2017; Xu et al., 2017; Lun et al., 2019). When raised in a germ-free environment, the *kd/kd* mice that spontaneously develop interstitial nephritis rarely display kidney disease (Hallman et al., 2006). In addition, CKD leads to gut dysbiosis with a reduction in the abundance of genera *Bacteroides* and *Lactobacillus* and an increase of *Enterobacteriaceae* (Fukuuchi et al., 2002; Vaziri et al., 2013; Kikuchi et al., 2017). This shift of gut flora drives a cascade of metabolic abnormalities, including uremic toxin production and inflammation, which ultimately leads to progressive kidney failure and CKD complications (Evenepoel et al., 2009; Kikuchi et al., 2017; Xu et al., 2017; Yang et al., 2018). The critical role of gut microbiota in CKD was further strengthened by the fact that reversing gut dysbiosis improves kidney function. For example, resistant starch alters the impaired gut microbial community and thus exert a renoprotective effect in CKD rats (Kieffer et al., 2016). Despite an increasing recognition of the importance of gut microbiota in CKD progression, few studies evaluate the response of gut microbiota to CKD drugs to examine its roles in mediating or affecting drug efficacy (Mishima et al., 2015; Mishima et al., 2018).

Currently, few therapeutic options are available to slow or prevent the progression of CKD, especially in China where many patients have turned to traditional Chinese medicines (TCM) for CKD treatment. TCM has a long history for treating CKD and is able to achieve recognizable improvement in patient outcomes, but its underlying mechanisms remain largely unknown. Several studies have suggested that gut microbiota might be one of the essential targets of TCM, which usually contains various bioactive components and may exert beneficial effects on the host through modulating the gut microbiota (Cardona et al., 2013; Chang et al., 2015; Wu et al., 2019). For instance, polysaccharides derived from *Ganoderma lucidum* and *Hirsutella sinensis* promote the growth of probiotics which contribute to weight control in mice (Chang et al., 2015; Wu et al., 2019). In contrast to TCM, a recent study showed that most of the commonly used FDA approved human-targeted drugs inhibit the growth of gut commensals (Maier et al., 2018). Moreover, a recent clinical study demonstrated that distinct gut microbial communities of type 2 diabetes patients under TCM and metformin treatment contribute to their difference in ameliorating insulin resistance and hyperlipidemia (Tong et al., 2018). These studies hint at a more positive role of gut microbiota in TCM than other drugs, given the rich array of the naturally derived components in TCM to promote recovery from gut dysbiosis associated with disease. However, so far, no study compares the effects of TCM and kidney-targeted drugs on gut microbiota to characterize the interactions between microbes and their links with clinical outcomes.

Here, we examined the responses of both the host and its gut microbiota to Jian-Pi-Yi-Shen (JPYS) decoction, a TCM containing eight Chinese herbs specially designed for CKD treatment, and to piperazine ferulate (PF), a kidney-targeted drug widely used for the treatment of CKD. Clinical parameters, whole blood hematological and histological analysis were employed to measure and compare treatment outcomes between the two drugs. We performed 16S rDNA sequencing to profile the gut microbiome of the CKD rats, and the rats treated with JPYS or PF. Differential microbiome responses were characterized by UniFrac beta-diversity and microbiome markers specific to the drugs. To understand interspecies interactions between the markers, and their correlations with treatment outcomes or microbiome functions, we constructed correlation network of the markers, together with clinical parameters and KEGG pathways inferred by PICRUSt. Network hubs forming by microbiome markers with dense connections were identified to pinpoint keystone genera that were driving the response to drugs and associated with clinical parameters or KEGG functions. At last, the identified keystone genera of drug response were validated by quantitative polymerase chain reaction (qPCR) for their differential abundance between CKD and the two drugs.

## Material and Methods

### Preparation of JPYS Decoction

The preparation of the JPYS decoction and quality control were conducted as described in our previous study (Chen et al., 2018). JPYS composes of eight herbs and the ratio of the respective herb is illustrated in **Table S1**. All the botanical names can be checked and validated using the Kew Medicinal plant names service (http://mpns.kew.org/mpns-portal/?_ga=1.111763972.1427522246.145907734). The decoction pieces of eight Chinese herbal medicines were purchased from Shenzhen Huahui Pharmaceutical Co., Ltd. Assurance of quality control for all the materials was validated according to the Chinese Pharmacopeia (Chinese Pharmacopoeia Committee, 2015). Briefly, all herbal ingredients were weighed (121.0 g in total per dose) and extracted with boiling water. The extract was dried to powder (66.7 g) and stored at -80°C until use. JPYS extract was chemically standardized and evaluated by HPLC-MS analysis, as indicated in **Figure S1**.

### Study Design and Animal Experiments

Male Sprague–Dawley rats weighting 160–180 g were allowed to acclimatize or four weeks prior to experimentation. All rats were housed at a density of five per cage, bred under controlled room temperature (20 ± 1°C) and humidity (70%) with 12/12-h light-dark cycle, and with free access to water and food. CKD was induced in 30 rats by a two-step 5/6 nephrectomy as previously described (Platt et al., 1952). Briefly, the first renal surgery involved electrocautery of the left kidney except for a 2-mm area around the hilum. A second renal surgery was performed two weeks later by double ligations of the renal hilum with silk suture and surgical excision of the right kidney. Ten rats underwent sham surgery were used as controls. After 16 weeks, the CKD rats were randomly divided into three groups (n = 10 per group), and received daily oral gavage with JPYS (10.89 mg/kg), PF (50 mg/kg) or water for 12 weeks, for each group respectively (Chen J. et al., 2019). The sham-operated rats received water daily. At the end of the experiment, urine and fecal samples were collected from rats before sacrificed. Blood samples and kidney tissues were collected for further studies. All animal works were approved by the Guangzhou University of Chinese Medicine Institutional Animal Care and Use Committee.

### Clinical Parameters, Whole Blood Hematological and Histological Analysis

Whole blood hematological parameters were tested with an ADVIA 2121i hematology (**Table S2**). Serum or urine biochemical parameters were determined by enzyme-linked immunosorbent assay (ELISA) or chemical tests using respective kits (**Table S2**) according to their manufacturers’ recommendations. Rat kidney were collected and fixed in 10% formaldehyde, embedded in paraffin, cutted into 4 μm sections, and stained with periodic acid-Schiff (PAS) or Masson’s trichrome staining (MTS). Histological images were obtained with a light microscope. 10 images were captured for each sample (**Figure S2**).

### DNA Extraction and 16S rRNA Gene Amplicon Sequencing

DNA was extracted from rats’ fecal samples with MOBIO PowerSoil^®^ DNA Isolation Kit (12888-100) and stored at -80°C in Tris-EDTA buffer solution until use. The V4 region of the 16S rRNA gene was polymerase chain reaction (PCR) amplified using barcoded universal primers (515F-806R) and sequenced with the Illumina MiSeq platform (2 × 250bp).

### 16S rDNA Sequence Data Analysis

Raw 16S rDNA sequencing reads were profiled using USEARCH (version 10.0) (Edgar, 2010). Briefly, sequencing quality of raw reads was inspected using FastQC (version 0.11.5) (https://www.bioinformatics.babraham.ac.uk/projects/fastqc/). Paired-end reads were joined and demultiplexed through the USEARCH pipeline. After that, primers were trimmed using cutadapt (version 1.16) (Martin, 2011). Operational taxonomic units (OTUs, 97% sequence similarity) were clustered and processed by using the USEARCH commands: “-cluster_otus”, “-otutab_trim”, and “-otutab_norm”, to remove low-abundance OTUs and normalized the processed OTU table to 5,000 reads. USEARCH commands “-alpha_div” and “-beta_div” were used to generate alpha- and beta-diversity profiles, including the number of observed OTUs, other alpha-diversity index and the UniFrac distances. Taxonomy was assigned to each OTU using the Ribosomal Database Project (RDP) classifier (Wang et al., 2007). PICRUSt (version 1.1.3) was used to predict functional pathway based on 16S rRNA gene sequencing data for each sample (Langille et al., 2013). From the software, the python scripts: “predict_metagenomes.py” and “categorize_by_function.py”, were used to generate KEGG profiles.

### Microbiome Markers, Correlation Network and Statistical Analysis

Statistical analysis was performed using the R software (version 3.6.0) and Python (version 3.5). Microbiome markers were selected by the Boruta algorithm (R package, Boruta, version 6.0.0), and bootstrapping (n = 5000) was used to assess the statistical significance of the selected markers, as indicated as “confirmed” in Boruta output (Kursa and Rudnicki, 2010). The false discovery rate (FDR) adjusted Wilcoxon rank-sum test was implemented to examine significant difference between groups relative abundance of taxa. Permutation multivariate analysis of variance (PERMANOVA, adonis in the vegan R package, version 2.5-6) (Oksanen et al., 2018) was conducted on UniFrac distance matrix and visualized by PCoA (R package, stats, version 3.6.0) (R CoreTeam, 2013). Genus-to-genus, genus-to-KEGG pathway and genus-to-clinical parameter correlation network was generated based on the Spearman correlation coefficient, and edges were filtered according to their FDR adjusted *p*-value. The network was constructed by the NetworkX python module (version 2.3), and only edges of correlation significance test *p*-value < 0.05 were plotted. Visualization of the network was performed within Cytoscape (version 3.7).

### qPCR Validation of Microbiome Markers From Fecal Samples

Relative abundance of the selected keystone microbiome markers identified by 16S rDNA sequencing were further measured by qPCR. Primers were designed with the Primer-BLAST tool based on the V4-region extracted from 16S rDNA sequences of the representative OTUs of the genera identified by Boruta, excepted for genera *Enterococcus* for which the primers was adopted from a previous study (Matsuda et al., 2009) (**Table S3**). Primer specificity was checked with Primer-BLAST and only species-specific primers were used for qPCR analysis. qPCR was performed with PowerUP SYBR Green master mix (Thermo Fisher Scientific, USA) on a BioRad CFX96 Real-Time PCR detection system. The cycling condition for qPCR reactions was 95°C for 2 min, 40 cycles of 95°C for 5 s, 60°C for 30 s. Melt curve analysis was performed to check amplification specificity. Total bacterial load was determined by qPCR with previously described universal bacterial primers (Ramseier et al., 2009). Relative abundance of each species was normalized to the total bacterial load and expressed as 2^-ΔCT^.The false discovery rate (FDR) adjusted Wilcoxon rank-sum test was performed to analyze qPCR results to examine their statistical significance of difference between groups.

## Results

Before biological investigation, JPYS was firstly chemically standardized. An HPLC-MS method was established to explore the chemical profile of JPYS extract and quantify the main ingredients in the extract. By using the respective individual standard, ten chemical markers were identified from JPYS extract (**Figure S1**) and the minimum amount in μg/g of dried extract was 100.52 for acteoside, 79.03 for calycosin 7-O-glucoside, 257.68 for liquiritin, 61.59 for rosmarinic acid, 518.72 for salvianolic acid A, 13.19 for astragaloside IV, 1377.14 for rhein, 3.45 for dioscin, 9.26 for atractylenolide I, and 0.19 for tanshinone IIA. The chemical analysis of JPYS extract here employed as the quality control method for the reproducibility of the below study.

**Figure 1:**
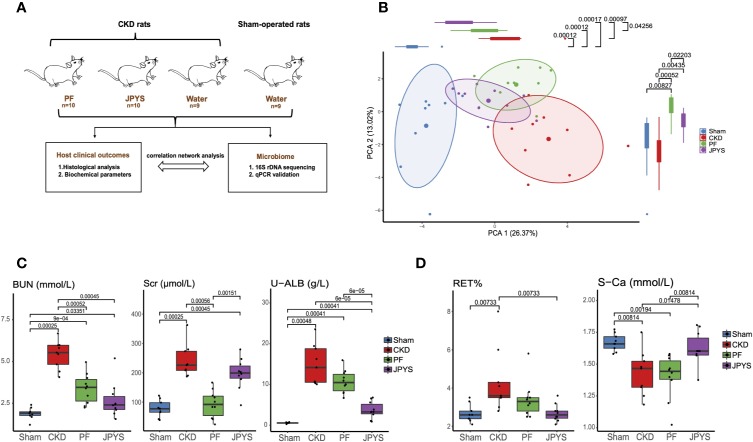
Distinct host responses to JPYS and PF in the CKD rats. **(A)** Study design to evaluate the responses of the CKD rats and their gut microbiota to JPYS or PF. **(B)** Principal component analysis (PCA) of clinical parameters of the sham-operated, CKD, JPYS and PF rats after 12 weeks of treatment (Sham: n = 9, CKD: n = 9, JPYS: n = 10 and PF: n = 10). Overall clinical variation of the four treatment groups was assessed along PC1 and PC2 respectively. **(C)** Variation of clinical parameters of kidney function in each group. **(D)** Variation of clinical parameters of CKD complications in each group. Statistical significance of variation between groups was tested by Wilcoxon rank-sum test with FDR correction. Only significant differences between groups (*p* < 0.05) were annotated. JPYS, Jian-Pi-Yi-Shen decoction; PF, Piperazine ferulate; CKD, chronic kidney disease; BUN, blood urea nitrogen; U-ALB, urinary albumin; RET, reticulocyte; S-Ca, serum calcium levels.

A total of 30 CKD rats along with 10 sham-operated rats were used in this study. Two rats died before the end of the experiment and were excluded from further analysis. The study design is provided in **Figure 1A**. Briefly, host clinical outcomes were evaluated by histological analysis of rat kidney tissue and clinical indicators of renal function or CKD complications. Gut microbiome were analyzed by 16S rDNA sequencing. Microbiome markers of drug response were identified and validated by qPCR. Network analysis of host clinical parameters and gut microbiota was performed to characterize the interactions between microorganisms and with the host.

### JPYS Achieved an Improved Treatment Outcome in Preventing the Development of CKD and Its Complications

To evaluate the effect of JPYS and PF on the progression of CKD and its complications in nephrectomized rats, we measured the cell composition of whole blood and 16 biochemical parameters relevant to renal function or CKD complications after 12 weeks of treatment. Principal component analysis (PCA) was performed to access the pattern of variation among host clinical phenotypes based on their clinical parameters. The clinical phenotypes of the CKD rats were significantly different from that of the sham-operated rats along the first principal component (PC1) (*p* = 0.00012), indicating a major component of phenotype variation related to CKD progression (**Figure 1B**). By assessing the treatment effect along this component, JPYS (*p* = 0.00097) significantly improved the kidney function as depicted by the right-to-left displacement of its PC1 location from CKD to the sham group (JPYS VS. CKD, *p* = 0.00097; JPYS vs. Sham, *p* = 0.00017), which was not observed for PF (PF vs. CKD, *p* > 0.05; PF vs. Sham, *p* = 0.00012). Dissimilarity of host responses to JPYS and PF was also reflected in PC2 (*p* = 0.0220). We next sought to identify the differences in individual clinical parameters between JPYS and PF treatment. To this end, we first compared the well-established urine and serum indicators of renal function, including serum creatinine (Scr), blood urea nitrogen (BUN) and urinary albumin (U-ALB). The level of BUN was significantly reduced in both the JPYS (*p* = 0.00045) and the PF (*p* = 0.0052) groups comparing to that of CKD rats without treatments (**Figure 1C**, **Table S2**). While JPYS had a better effect on reducing the level of U-ALB (*p* = 0.00006), decrease in the Scr level (*p* = 0.00056) was only significant in the PF group (**Figure 1C**, **Table S2**). We further evaluated the impact of the two drugs on indicators of CKD complications. Our results showed that only JPYS was able to significantly restore the blood reticulocyte (RET) (*p* = 0.00733) and serum calcium levels (S-Ca) (*p* = 0.01478) in CKD rats (**Figure 1D**, **Table S2**).These results indicated JPYS achieved an improved overall treatment outcome of CKD which can be explained by clinical parameters responded to JPYS differently from PF.

### Microbiome Responses Distinct to JPYS and PF Were Identified at Various Taxonomic Levels

**Figure 2:**
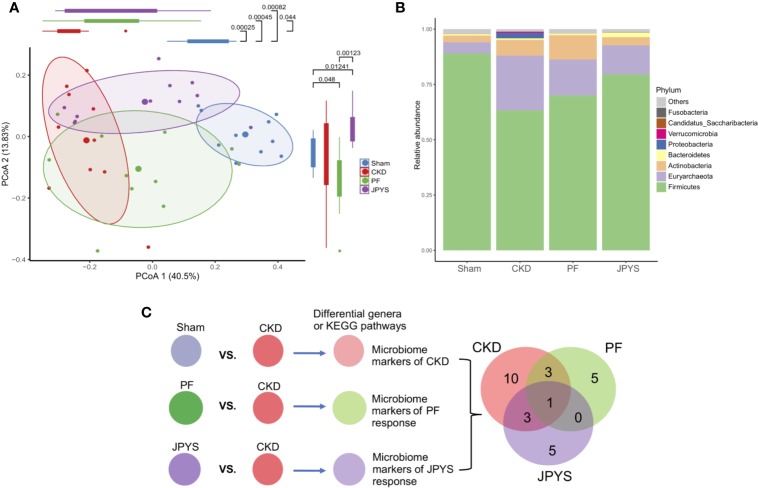
Distinct responses of gut microbiome to JPYS and PF in the CKD rats. **(A)** Weighted UniFrac-based principal coordinates analysis (PCoA) of the gut microbiome of the sham-operated, CKD, JPYS, and PF rats after 12 weeks of treatment. Statistical significance of variation of the microbiomes was assessed along PC1 and PC2 respectively, tested by Wilcoxon rank-sum test with FDR correction. **(B)** Relative abundances of phyla across the four treatment groups. **(C)** Selection of genus-level microbiome markers by the Boruta algorithm and Venn diagram of the selected markers that are common or specific to disease or treatment. JPYS, Jian-Pi-Yi-Shen decoction; PF, Piperazine ferulate; CKD, chronic kidney disease; PC1, first principal component; FDR, false discovery rate.

Responses of gut microbiota to medication might mediate or influence host clinical outcomes (Wu et al., 2017; Maier et al., 2018). To characterize the gut microbiota variations among the hosts of the JPYS and PF groups, we profiled their microbial communities by 16S rDNA sequencing and examined their weighted UniFrac beta-diversity using principal coordinates analysis (PCoA). The gut microbiome was significantly different between the rats of the CKD group and the sham group, as depicted by PC1 (**Figure 2A**). A concordant shift of the microbial profiles of CKD rats treated with either PF or JPYS along PC1 can be observed, although not significant in the JPYS group (PF, mean ± sd,-0.07397 ± 0.17550, *p* = 0.0440; JPYS, mean ± sd, -0.01601 ± 0.21090, *p* > 0.05). Significant differences of gut microbiota between JPYS and PF can be observed along PC2 (*p* < 0.001), but might not be related to the microbiome variation in CKD (CKD vs. Sham, *p* > 0.05). We then sought to compare the gut microbiota to JPYS and PF to identify broader taxonomic responses at the phylum level. The relative abundance of Firmicutes was significantly decreased in CKD rats (*p* = 0.00025),while relative abundances of Euryarchaeota (*p* = 0.00740), Proteobacteria (*p* = 0.01587) and Actinobacteria (*p* = 0.02120) were significantly higher than that of the sham-operated rats (**Figure 2B**, **Table S4**). In response to treatment, PF significantly decreased the level of Proteobacteria induced by CKD (*p* = 0.01587), while JPYS restored the levels of Firmicutes (*p* = 0.04404) and Actinobacteria (*p* = 0.02582) (**Figure 2B**, **Table S4**). We further pinpointed the differential microbiome markers at the genus level by the Boruta feature selection algorithm. A total of 17 genera significantly differed between the sham-operated rats and the CKD rats. Among these microbiome markers of CKD, only one responded to both JPYS and PF (**Figure 2C**); three responded specifically to JPYS; and another three responded specifically to PF. Besides, five non-CKD-related microbiome markers were found to respond only to JPYS and another five responded only to PF. We designated these 10 genera markers as either JPYS- or PF-only marker in this study for further examination. Taken together, these findings revealed various levels of gut microbiota responses distinct to JPYS, which are significantly differed from that to PF, and might be further linked with particular microbial functions and clinical phenotypes of the host.

### Keystone Microbiome Markers of Drug Response Were Revealed and Linked With Clinical Phenotypes

To understand the interactions between the identified genera and their association with host responses to JPYS and PF, we first constructed a correlation network of the genera, and then evaluated genus-host interaction by correlation analysis of the genera and host clinical parameters. All the genera selected by Boruta were used for correlation analysis and connections were drawn only for those correlations above a given threshold (correlation coefficient |ρ| > 0.6) for network building and visualization.

**Figure 3:**
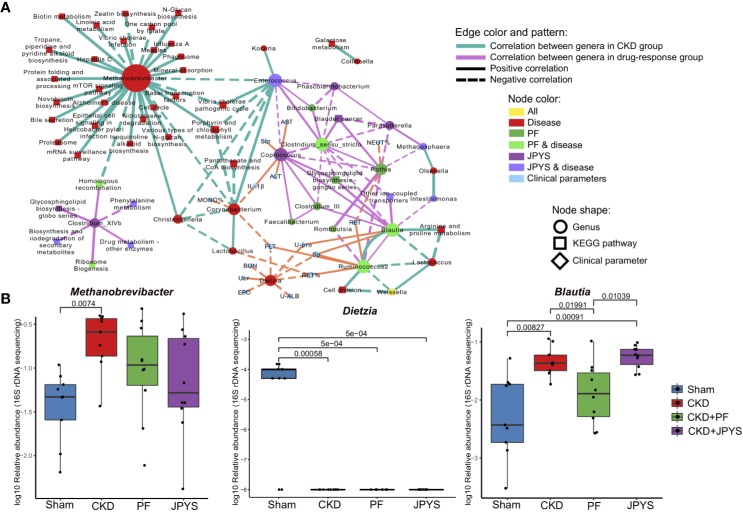
Gut microbiome markers correlated with host clinical outcomes. **(A)** Correlation network of microbiome marker, clinical parameter, and KEGG pathway. Circles represent markers of genus; Squares represent KEGG pathways; and diamonds represent clinical parameters. Node colors indicate treatment groups. Node sizes are scaled according to their degrees of connections. The larger the node is, the more connections it has. Two nodes are connected if their spearman correlation is significant (*p* < 0.05, FDR-corrected) between genera, or between genera and KEGG pathways. Connections are drawn between genera and clinical parameters only when their spearman correlation is significant (*p* < 0.05, FDR-corrected) and the absolute of correlation coefficient is larger than 0.6. Thickness of edges between nodes is scaled according the absolute value of spearman correlation coefficient. Edge colors indicate correlations between genera (or between genera and KEGG pathways) in different groups (green: CKD group; pink: treat groups of PF and JPYS). Orange edges represent correlations between genera and clinical parameters. Dotted and solid lines indicate negative or positive correlations respectively. **(B)** Relative abundances of keystone genus-level microbiome markers in each group. Keystone markers are manually selected from the network hubs, with regard to their interspecies interactions and clinical associations. Variations between groups were tested by Wilcoxon rank-sum test with FDR correction. Only significant differences between groups (*p* < 0.05) were annotated. FDR, false discovery rate; JPYS, Jian-Pi-Yi-Shen decoction; PF, Piperazine ferulate; CKD, chronic kidney disease.

Microbiome markers of CKD formed different subnetworks of connections with the markers of JPYS or PF (**Figure 3A**). Several markers of CKD had no significant correlation directly to either JPYS or PF, including *Methanobrevibacter*, *Corynebacterium*, *Lactobacillus*, *Dietzia*, and *Lactococcus*, representing microbiome signatures of CKD without direct response to the drugs. However, there was a network hub formed by *Enterococcus*, a marker of both CKD and JPYS, with connections to both *Methanobrevibacter* and *Corynebacterium*, which might mediate the effect of JPYS through inter-species interactions. Another two network hubs were made by *Blautia* and *Ruminococcus2*, markers of both CKD and PF, connected with the CKD-related *Lactococcus* and PF-only of *Clostridium_III*, reflecting possible drug responses and medication effects of PF associated with these microbiome markers. There was only one CKD-related genus, *Weissella*, which responded to both JPYS and PF.

The JPYS-only marker *Coprococcus* served as a hub correlated with many genera, such as the PF-only marker *Feaclibacterium* and *Clostridium_III*, and also correlated with two disease markers of CKD with drug response, such as *Enterococcus* and *Clostridium_sensu_stricto*. Furthermore, this hub was associated with multiple clinical parameters, including Scr (a well-established indicator of kidney function) and IL-1β (a central element of kidney inflammation), which suggest that the response of the genus may contribute to host clinical outcomes. Additionally, these parameters also include alanine aminotransferase (ALT) and aspartate aminotransferase (AST), which are indictors of liver function. Taken together, these connections indicate that this microbiome markers might serve as a keystone taxon coordinating interspecies interactions and their responses to drugs, and mediating drug effects on clinical outcomes. In contrast, the CKD-related genus *Dietzia* negatively correlated to several clinical parameters, but without direct response to the two drugs. There were also microbiome makers independent from other genera, representing distinct microbiome signatures of drug response. For instance, the JPYS-only marker *Clostridium_XIVb* connected with several KEGG pathways, including phenylalanine metabolism, glycosphingolipid biosynthesis globo series, and biosynthesis and biodegradation of secondary metabolites. These pathways have been shown to be associated with CKD development and progression (Kopple, 2007; Mather and Siskind, 2011).

We then examined the response of the identified microbiome markers to drugs in terms of their changes in relative abundance between disease and drug groups, especially those of network hubs and of close associations with clinical parameters or pathways. As shown in **Figure 3B**, the relative abundance of *Methanobrevibacter* (CKD vs. Sham, *p* = 0.00740) significantly increased, while the level of *Dietzia* (CKD vs. Sham, *p* = 0.00058) significantly reduced in CKD rats. These two genera did not restore by neither JPYS or PF and negatively correlated with several clinical parameters, and may act as targets for microbiota-based therapy.The relative abundance of the PF responder *Blautia* was significantly higher in CKD rats and restored by PF (CKD vs. PF, *p* = 0.01991, **Figure 3B**, **Table S5**). By combining the network context and their changes in disease or treatment groups, the identified genera allow us to pinpoint candidate biomarkers to evaluate the responses of gut mcirobiota to the two drugs tested, augmented with evidence from previous studies (**Table S5**).

### qPCR Validated the Changes of Keystone Microbiome Markers Associated With Distinct Drug Response

**Figure 4:**
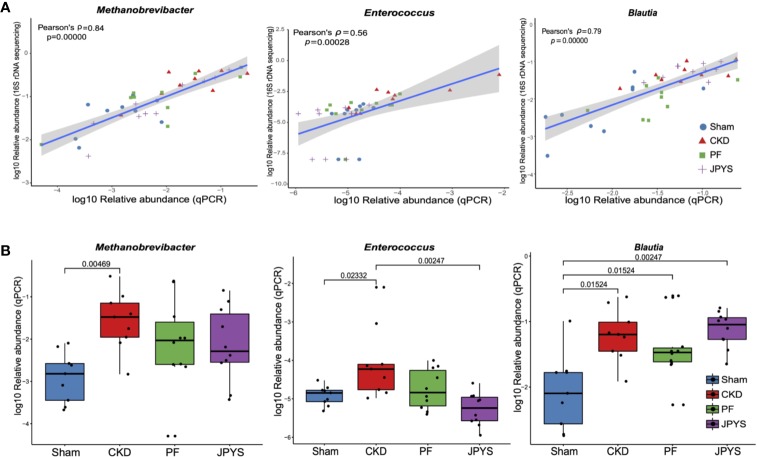
qPCR validation of the identified keystone microbiome markers. **(A)** Pearson correlation between relative abundance of the genera *Methanobrevibacter, Enterococcus* and *Blautia* from 16S rDNA sequencing and qPCR data. Colors and shapes of the plot symbols indicate samples from different treatment groups. Shaded area represents the 95% confidence interval for the regression line. **(B)** Relative abundance of the genera *Methanobrevibacter, Enterococcus*, and *Blautia* across treatment groups, as detected by qPCR. Variations between groups were tested by Wilcoxon rank-sum test with FDR correction. Only significant differences between groups (*p* < 0.05) were annotated. FDR, false discovery rate.

To validate the keystone microbiome markers of distinct drug response, we further performed species-specific qPCR assay to assess their differential abundance among disease or treatment groups. The 25 genera identified as disease or drug response markers, were subjected to qPCR validation. To allow for qPCR validation, 78 OTUs from these genera were extracted and filtered to remove OTUs that are not associated with disease or drugs (58 OTUs filtered). The remaining 20 OTUs (belonging to 11 genera) were further subjected to the design of species-specific primers, and only five genera have representative OTUs that passed the check of primer specificity with Primer-BLAST. Together with *Enterococcus*, for which we were able to obtain primers from previous studies, a total of six genera were finally validated with qPCR to confirm the results from 16S rDNA sequencing. For the others, due to the lack of full-length sequences of their 16S rRNA genes with the current V4-region-based 16S rDNA amplicon sequencing protocol, we were unable to design their species-specific primers for qPCR validation. As shown in **Figure 4A**, the relative abundances of the representative OTUs of genus *Methanobrevibacter* (*p* = 3.75e-11, Pearson’s ρ = 0.84)*, Enterococcus* (*p* = 0.00028, Pearson’s ρ = 0.56), and *Blautia* (*p* = 3.55e-09, Pearson’s ρ = 0.79) were significantly correlated between qPCR and 16S rDNA sequencing. Significant correlation between qPCR and 16S rDNA sequencing were also obsevered in genera *Ruminococcus2* (*p* = 0.00013, Pearson’s ρ = 0.58)*, Clostridium_sensu_stricto* (*p* = 6.02e-07, Pearson’s ρ = 0.71) and *Phascolarctobacterium* (*p* = 2.17e-10, Pearson’s ρ= 0.82) as shown in **Figure S3**. After removing the groups of outliers, the correlations for Enterococcus and Ruminococcus2 are still significant, suggesting the correlations are not driven by the outliers (**Figure S4**). qPCR also confirmed the significant enrichment of the reprehensive OTU of genus *Methanobrevibacter* in CKD rats, which was consistent with that of *Methanobrevibacter* as we obtained by 16S rDNA sequencing (**Figure 4B**, **Table S5**). Consistent changes in the relative abundance of genera *Enterococcus* and *Blautia* was also obsevered (**Figure 4B**, **Table S5**). We also perform association analysis between the parameters described in **Figure 3A** and the relative abundances assessed with qPCR (**Figure S5**). The result shows that all the associations that present are confirmed by qPCR. In detail, the regression plots and their p-values for the relative abundances from 16S rDNA sequencing are confirmed by the same analysis from qPCR; and the 95% confidence intervals of the correlation coefficient are also compatible to each other.

## Discussion

The development and progression of CKD has been associated with host gut microbiota, and possible mechanical links *via* host-microbiome interactions were proposed. It is likely that distinct responses of gut microbiome that are specifically to different drugs might affect or mediate their treatment effects. In this study, we showed that both JPYS and PF prevented kidney damage (**Figure S2**) and significantly decreased the levels of BUN and TNF-α (**Figure 1C**, **Table S2**). But there were also different clinical outcomes for the two drugs, with the level of Scr significantly decreased in PF group only and the level of U-ALB significantly decreased in JPYS only (**Figure 1C**, **Table S2**). JPYS also displayed a better effect in restoring the levels of RET and S-Ca which may prevent CKD-associated anemia and mineral bone diseases (**Figure 1D**, **Table S2**) (Tsagalis, 2011; Hou et al., 2018). This is consistent with our previous study which showed that JPYS was able to alleviate renal anemia in CKD rats (Chen J. et al., 2019). Taken together, both the two drugs improved kidney function, but JPYS achieved a better treatment outcome in delaying the progression of CKD complications.

Sequencing and analysis of the gut microbiota of the CKD rats revealed distinct microbiome profiles from that of the sham-operated rats. Particularly, the relative abundances of phyla Proteobacteria and Actinobacteria were significantly higher in CKD rats, which are in line with previous studies that demonstrated the enrichment of Proteobacteria in CKD patients and CKD rats (Lun et al., 2019). Members of Proteobacteria are mainly opportunistic pathogens and the expansion of this phylum usually indicates an imbalanced and unstable gut microbial community of the host (Shin et al., 2015). Our study showed that both the two drugs were able to reduce the relative abundance of Proteobacteria in CKD rats. Therefore, as a treatment outcome with respect to the gut microbiota, both JPYS and PF could result in a healthier gut microbial community than that of the CKD rats. Furthermore, JPYS outweighs PF in restoring gut microbiota with regard to Actinobacteria, given that the change of Actinobacteria caused by CKD varies among studies and might be associated with the stage of CKD (Vaziri et al., 2013; Xu et al., 2017; Li et al., 2019).

An array of microbiome markers of CKD and drug response were identified and validated by qPCR in this study. In CKD rats, we found that the level of some opportunistic pathogens, including the CKD-markers of *Enterococcus, Weissella*, and *Clostridium_sensu_stricto*, were increased, while some host-beneficial microbes, such as *Lactococcus*, was decreased (**Table S5**), which are consistent with previous studies (Jiang et al., 2017; Kramer et al., 2018; Yang et al., 2018).

JPYS and PF each had distinct gut microbiota response as captured by their specific microbiome makers, with the exception of the CKD-related genus *Weissella*, which responded to both drugs. It is worth noting that, both JPYS and PF resulted in the increase of a group of butyrate producing genera. In the JPYS group, buryrate producing genera *Coprococcus*, *Phascolarctobacterium, Parasutterella* and *Clostridium_XlVb* were enriched, while PF increased other butyrate producing genera, such as *Bifidobacterium, Clostridium_sensu_stricto, Romboutsia, Blautia*, and *Ruminococcus2*. Butyrate producing bacteria were shown to be significantly enriched in healthy controls and decreased in ESRD patients (Jiang et al., 2017). The accumulation of short-chain fatty acids (SCFAs) and interaction between butyrate producing microorganisms may play a central role in medicating the treatment outcomes of CKD. However, further metabolomic studies on host and gut microbiota are needed to reveal the potential effect of JPYS and PF on SCFAs.

Integrative network analysis of microbiome markers of drug response and clinical parameters of host treatment outcomes allowed us to link interspecies interactions with their clinical phenotypes in detail. For instance, one of the network hubs formed by the JPYS-only marker *Coprococcus* also closely connected with many clinical parameters, such as Scr, an well-established clinical indicator of kidney function, hinting at a possible role of this genus on JPYS-specific clinical outcomes. Moreover, *Coprococcus* is often considered to be beneficial to host as this genus significantly decreased in many diseases, including inflammatory bowel disease, Parkinson’s disease and CKD (Rajilic-Stojanovic et al., 2011; Keshavarzian et al., 2015; Jiang et al., 2017), suggesting a possible route to mediate the treatment effect *via* modulating this keystone genus. Another network hub is formed by the PF responder *Blautia*, which was previously showed increased in CKD rats and end-stage renal disease patients on haemodialysis (Stadlbauer et al., 2017; Chen J. et al., 2019), suggesting a possible link of this genus with PF-specific clinical outcomes. Despite not directly connected with other microbiome markers, *Clostridium_XIVb* was connected with several CKD associated KEGG pathways. One of these pathways, the phenylalanine metabolism pathway was reported to be involved in CKD development and progression (Kopple, 2007). Degradation of phenylalanine by *Clostridium* leads to accumulation of uremic toxins, such as *p-*cresol and indole (Evenepoel et al., 2009). Therefore, the observed decreased abundance of *Clostridium_XIVb* by JPYS might reduce the burden of kidney disease.

In conclusion, our study showed that JPYS and PF had distinct clinical effect on CKD rats and their gut microbiota. The strong correlations between the gut microbiome markers, clinical parameters and molecular pathways hint at possible ways in which the identified keystone genus could be linked to the clinical outcomes under drug-specific microbiome responses. Though we have confirmed the identified microbiome markers by qPCR, further study is needed to culture and test the microbes to demonstrate their mechanism in mediating or affecting the drug effects based on the clues obtained in this study.

## Data Availability Statement

The 16S rDNA sequencing datasets for this study are available on the NCBI BioProject under the accession number PRJNA591716 (https://www.ncbi.nlm.nih.gov/sra/PRJNA591716).

## Ethics Statement

The animal study was reviewed and approved by the Guangzhou University of Chinese Medicine Institutional Animal Care and Use Committee.

## Author Contributions

SL, JC, and XL designed the study. FW, SH, and LZ participated in the animal experiments and FW finished the histological analysis. HZ and ML designed the microbiome data analysis. LZ, SC, XY, HZ, and ML participated in data analysis and interpretation of the results and prepared Figures. LZ, SC, and ML wrote the paper, contributed to literature search and data interpretation. HZ and G-PZ revised the manuscript. All authors read and approved the final version of manuscript.

## Funding

This research was supported by Natural Science Foundation of China (81804052 and 81973602), Natural Science Foundation of Guangdong Province (2018A030313305), and the Science Technology and Innovation Committee of Shenzhen Municipality grant (ZDSYS201606081515458). This work was also funded by Shenzhen Science and Technology Innovation Committee (Basic Science Research Grant JCYJ20170818154941048 to ZHOU Haokui) and Traditional Chinese Medicine Bureau of Guangdong Province (20201320).

## Conflict of Interest

The authors declare that the research was conducted in the absence of any commercial or financial relationships that could be construed as a potential conflict of interest.

## Supplementary Material

Figure S1 HPLC-MS chromatogram of JPYS extract. Equipment type: Shimadzu LCMS-8045; chromatographic column: Thermo Scientific Hypersil GOLD (150 × 4.6 mm, 3 μm); flow rate: 0.4ml/min; oven temperature: 35°C; mobile phase condition: the proportion of containing 0.1% formic acid **(A)** and acetonitrile **(B)** was as follows: 0–20 min, 28%-40% B; 20-25 min, 40%-70% B; 25-26 min, 70%-90% B; 26-36 min, 90%-90% B; 36-37 min, 90%-28% B. A Shimadzu mass spectrum (LCMS-8045) equipped with an ESI ion source was operated in positive and negative modes, and the multiple reaction monitoring was used. The detector voltagewas 1.74 kV; source temperature, 300°C; source voltage, 4 kV; nebulizer gas flow, 3 L/min; dry gas flow, 10 L/min; heater gas flow, 10 L/min. Air was used as heater gas at a flow rate of 10 L/min. Shimadzu Mass workstation software was used for data acquisition and processing. The denotation peaks 1-10 were acteoside (1), calycosin 7-O-glucoside (2), liquiritin (3), rosmarinic acid (4), salvianolic acid A (5), astragaloside IV (6), rhein (7), dioscin (8), atractylenolide I (9), tanshinone IIA (10). Representative chromatograms are shown, n = 3.

Figure S2 JPYS and PF prevented kidney damage. Representative histological images of periodic acid-Schiff (PAS, Scale bar = 10 μm) and Masson’s trichrome staining (MTS, Scale bar = 100 μm) of kidney sections from CKD rats.

Figure S3 qPCR validation of the identified microbiome markers. (a) Pearson correlation between relative abundance of the genera *Ruminococcus2, Clostridium_sensu_stricto* and *Phascolarctobacterium* from 16S rDNA sequencing and qPCR data. Colors and shapes of the plot symbols indicate samples from different treatment groups. Shaded area represents the 95% confidence interval for the regression line. (b) Relative abundance of the genera *Ruminococcus2, Clostridium_sensu_stricto* and *Phascolarctobacterium* across treatment groups, as detected by qPCR. Variations between groups were tested by Wilcoxon rank-sum test with FDR correction. Only significant differences between groups (*p* < 0.05) were annotated.

Figure S4 qPCR validation of the identified microbiome markers (relative abundance of zeros removed). Pearson correlation between the relative abundances assessed with 16S rDNA sequencing and qPCR, for the genera *Ruminococcus2* and *Enterococcus*. Colors and shapes of the plot indicate samples from different treatment groups. Shaded area represents the 95% confidence interval for the regression line.

Figure S5 Correlation between the clinical parameters described in **Figure 3A** and the relative abundances of microbiome markers. Regressions are shown between clinical parameters and the relative abundances of microbiome markers assessed with 16S rDNA sequencing (first column), or qPCR (second column). The third column shows the correlation coefficients with 95% confidence intervals for the regressions, accordingly.

Table S1 Herbal ingredients and propotion in JPYS.

Table S2 Details of clinical parameters tested and whole blood analysis in this study.

Table S3 Primers for qPCR analysis.

Table S4 Relative abundances of phyla across four groups.

Table S5 Microbiome markers identified by Boruta.
