# Supplementary figures and images for: Distinct Responses of Gut Microbiota to Jian-Pi-Yi-Shen Decoction Are Associated With Improved Clinical Outcomes in 5/6 Nephrectomized Rats
Source: Front Pharmacol. 2020 May 6;11:604. doi: 10.3389/fphar.2020.00604 (PMC7219274; doi:10.3389/fphar.2020.00604)

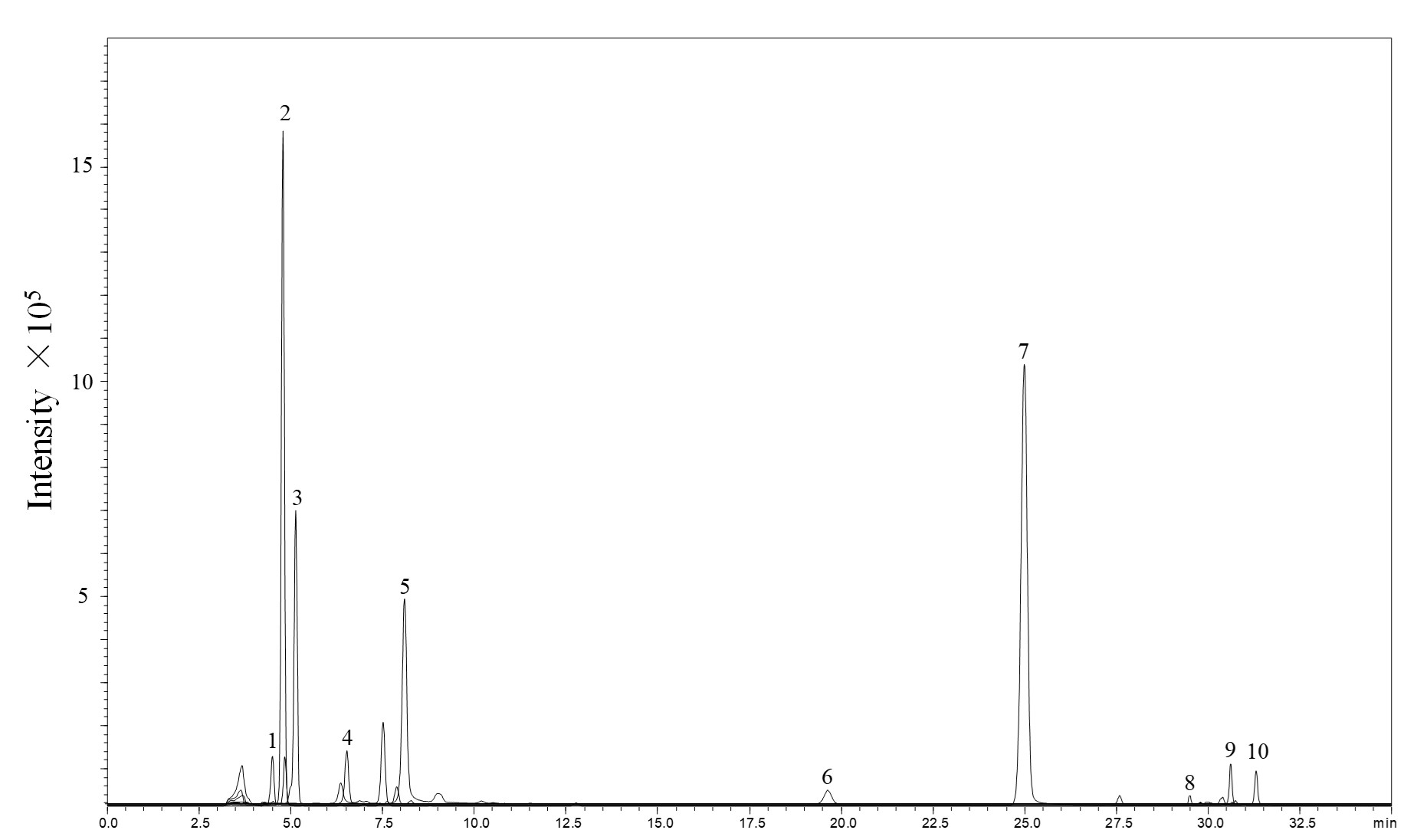

Supplement: Figure S1 — HPLC-MS chromatogram of JPYS extract. Equipment type: Shimadzu LCMS-8045; chromatographic column: Thermo Scientific Hypersil GOLD (150 × 4.6 mm, 3 μm); flow rate: 0.4ml/min; oven temperature: 35°C; mobile phase condition: the proportion of containing 0.1% formic acid (A) and acetonitrile (B) was as follows: 0–20 min, 28%-40% B; 20-25 min, 40%-70% B; 25-26 min, 70%-90% B; 26-36 min, 90%-90% B; 36-37 min, 90%-28% B. A Shimadzu mass spectrum (LCMS-8045) equipped with an ESI ion source was operated in positive and negative modes, and the multiple reaction monitoring was used. The detector voltagewas 1.74 kV; source temperature, 300°C; source voltage, 4 kV; nebulizer gas flow, 3 L/min; dry gas flow, 10 L/min; heater gas flow, 10 L/min. Air was used as heater gas at a flow rate of 10 L/min. Shimadzu Mass workstation software was used for data acquisition and processing. The denotation peaks 1-10 were acteoside (1), calycosin 7-O-glucoside (2), liquiritin (3), rosmarinic acid (4), salvianolic acid A (5), astragaloside IV (6), rhein (7), dioscin (8), atractylenolide I (9), tanshinone IIA (10). Representative chromatograms are shown, n = 3. [file Image_1.jpeg]

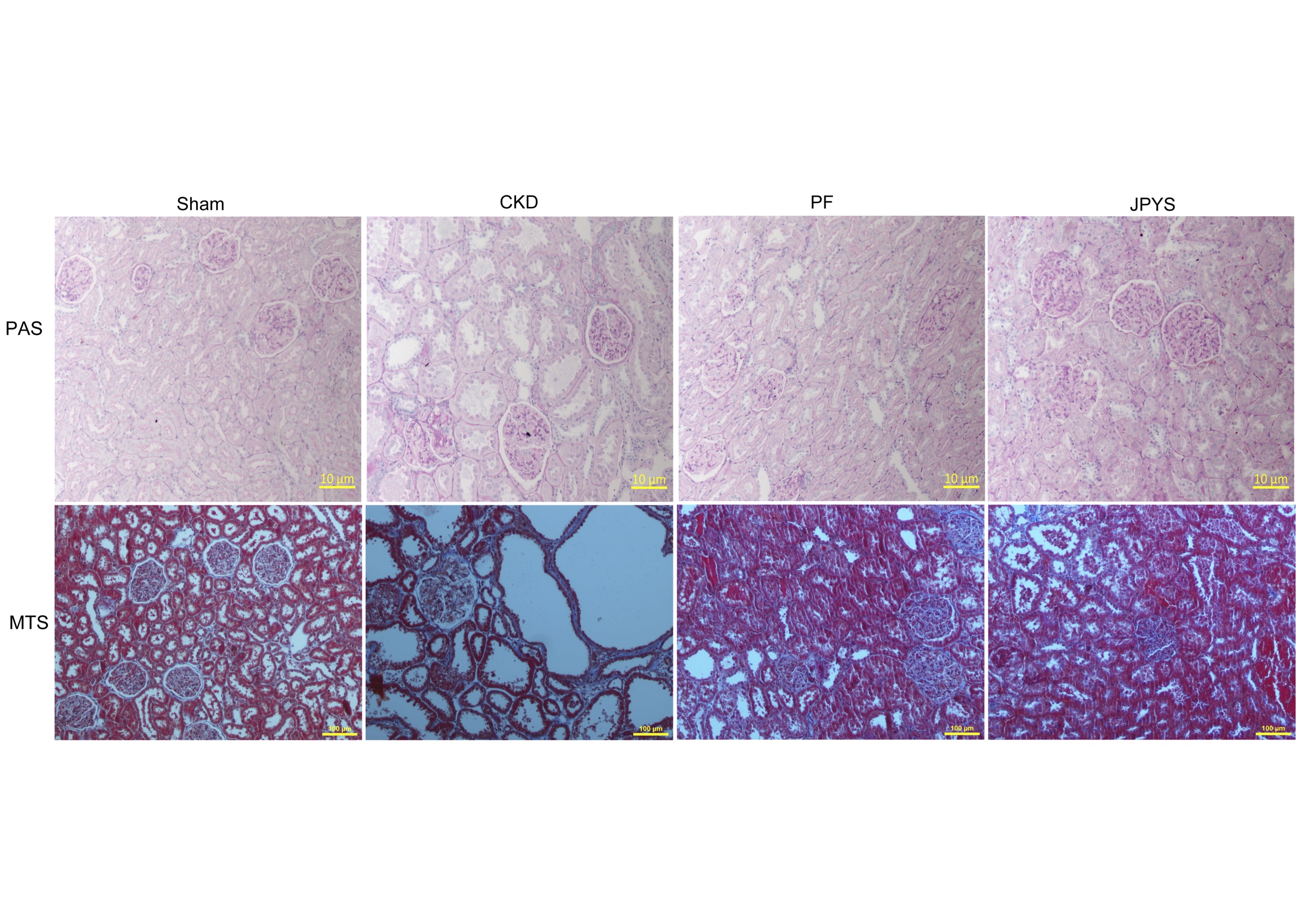

Supplement: Figure S2 — JPYS and PF prevented kidney damage. Representative histological images of periodic acid-Schiff (PAS, Scale bar = 10 μm) and Masson’s trichrome staining (MTS, Scale bar = 100 μm) of kidney sections from CKD rats. [file Image_2.jpeg]

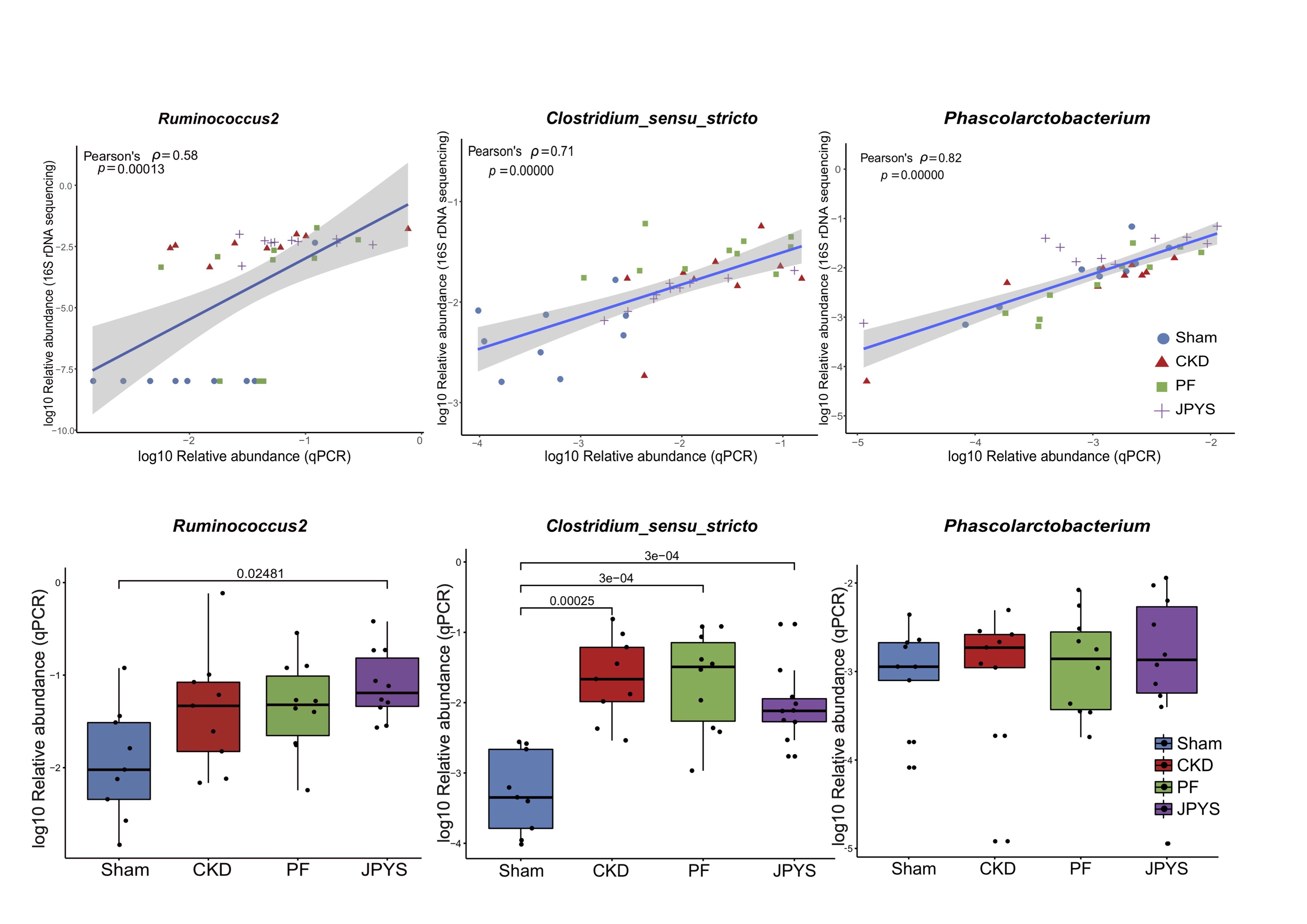

Supplement: Figure S3 — qPCR validation of the identified microbiome markers. (a) Pearson correlation between relative abundance of the genera Ruminococcus2, Clostridium_sensu_stricto and Phascolarctobacterium from 16S rDNA sequencing and qPCR data. Colors and shapes of the plot symbols indicate samples from different treatment groups. Shaded area represents the 95% confidence interval for the regression line. (b) Relative abundance of the genera Ruminococcus2, Clostridium_sensu_stricto and Phascolarctobacterium across treatment groups, as detected by qPCR. Variations between groups were tested by Wilcoxon rank-sum test with FDR correction. Only significant differences between groups (p < 0.05) were annotated. [file Image_3.jpg]

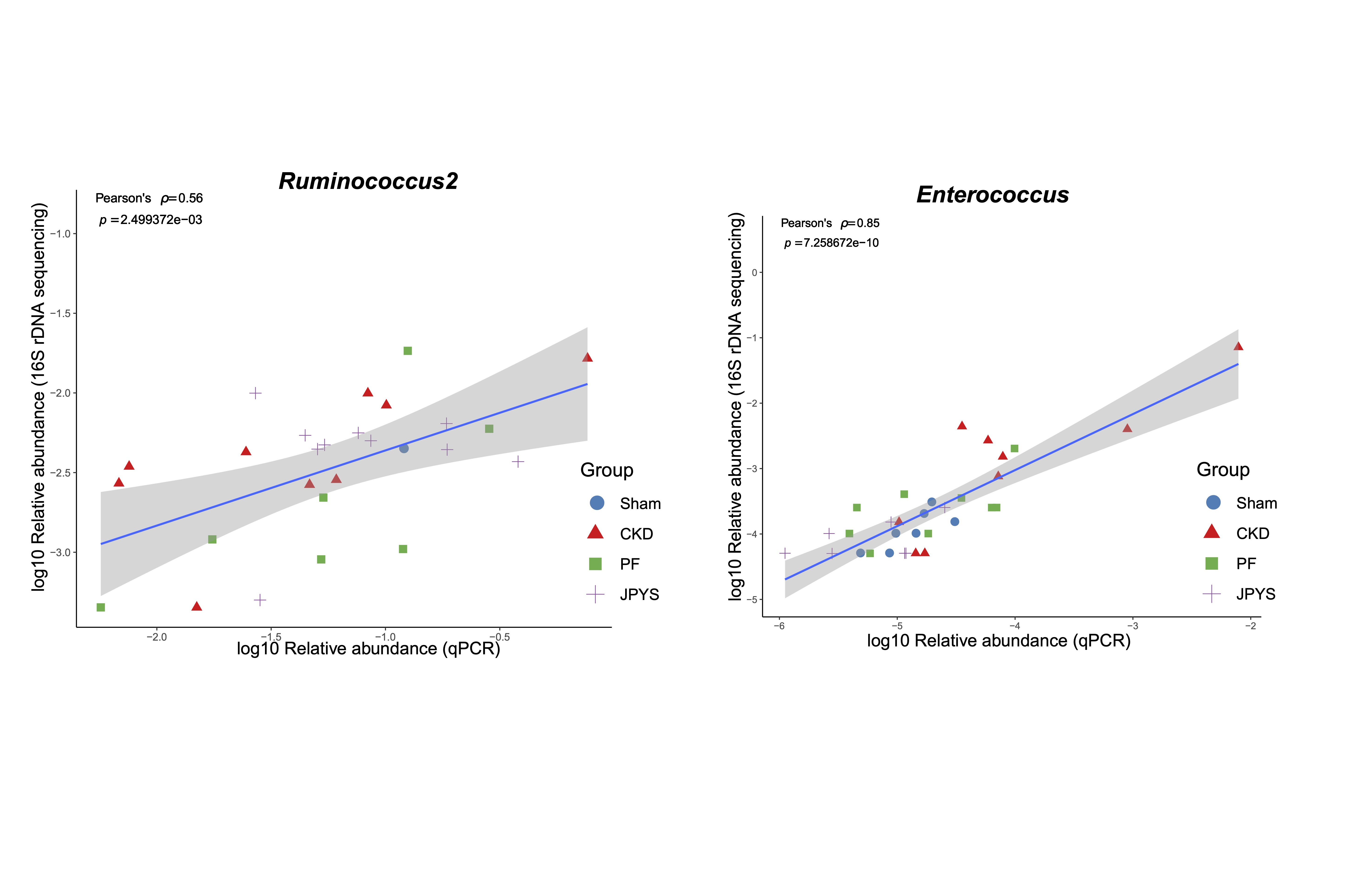

Supplement: Figure S4 — qPCR validation of the identified microbiome markers (relative abundance of zeros removed). Pearson correlation between the relative abundances assessed with 16S rDNA sequencing and qPCR, for the genera Ruminococcus2 and Enterococcus. Colors and shapes of the plot indicate samples from different treatment groups. Shaded area represents the 95% confidence interval for the regression line. [file Image_4.jpg]

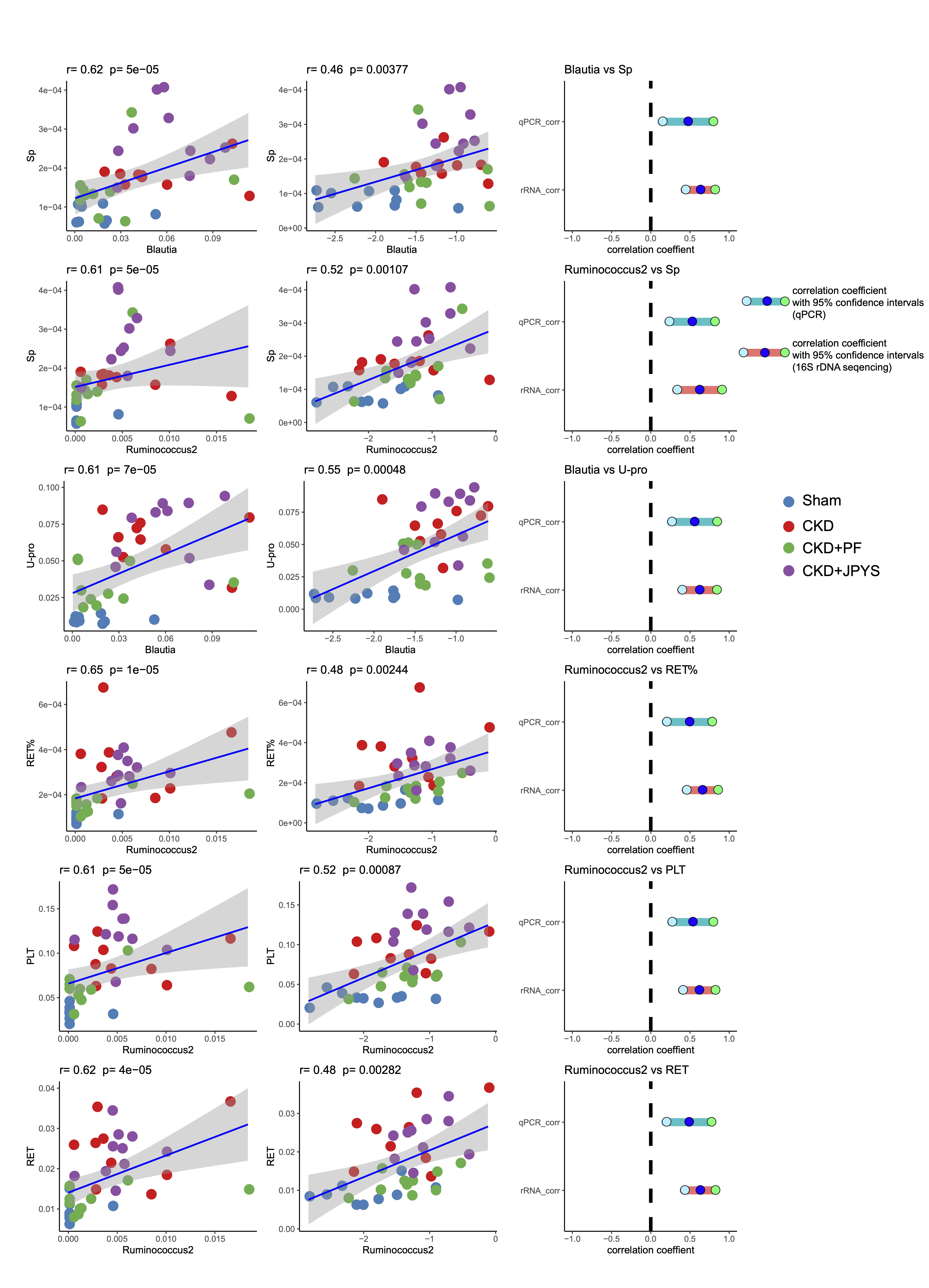

Supplement: Figure S5 — Correlation between the clinical parameters described in Figure 3A and the relative abundances of microbiome markers. Regressions are shown between clinical parameters and the relative abundances of microbiome markers assessed with 16S rDNA sequencing (first column), or qPCR (second column). The third column shows the correlation coefficients with 95% confidence intervals for the regressions, accordingly. [file Image_5.jpg]
